# Fatal Case of Heartland Virus Disease Acquired in the Mid-Atlantic Region, United States

**DOI:** 10.3201/eid2905.221488

**Published:** 2023-05

**Authors:** Sichen Liu, Suraj Kannan, Monica Meeks, Sandra Sanchez, Kyle W. Girone, James C. Broyhill, Roosecelis Brasil Martines, Joshua Bernick, Lori Flammia, Julia Murphy, Susan L. Hills, Kristen L. Burkhalter, Janeen J. Laven, David Gaines, Christopher J. Hoffmann

**Affiliations:** National Institute of Allergy and Infectious Diseases, Bethesda, Maryland, USA (S. Liu);; Johns Hopkins University School of Medicine, Baltimore, Maryland, USA (S. Kannan, M. Meeks, S. Sanchez, C.J. Hoffman);; Virginia Department of Health, Richmond, Virginia, USA (K.W. Girone, J.C. Broyhill, J. Bernick, L. Flammia, J. Murphy, D. Gaines);; Centers for Disease Control and Prevention, Atlanta, Georgia, USA (R.B. Martines);; Centers for Disease Control and Prevention, Fort Collins, Colorado, USA (S.L. Hills, K.L. Burkhalter, J.J. Laven)

**Keywords:** Heartland virus, viruses, zoonoses, vector-borne infections, parasites, emerging infections, tick-borne illness, hemophagocytic lymphohistiocytosis, Virginia, mid-Atlantic, United States

## Abstract

Heartland virus (HRTV) disease is an emerging tickborne illness in the midwestern and southern United States. We describe a reported fatal case of HRTV infection in the Maryland and Virginia region, states not widely recognized to have human HRTV disease cases. The range of HRTV could be expanding in the United States.

Heartland virus (HRTV) is a bandavirus spread by *Amblyomma americanum* (lone star) ticks in the midwestern and southern United States (*1*). Many cases of HRTV infection have been characterized by severe illness or death, mostly among men >50 years of age with multiple underlying conditions (*1*–*7*). HRTV infection in humans typically manifests as a nonspecific febrile illness characterized by malaise, myalgias, arthralgias, and gastrointestinal distress, along with thrombocytopenia, leukopenia, hyponatremia, and elevated liver transaminases (*3*). Most reported hospitalized patients recover, but deaths have occurred and have been associated with secondary hemophagocytic lymphohistiocytosis (HLH) (*4*,*5*).

Since HRTV was discovered in 2009 in Missouri, USA, human HRTV disease cases have also been reported in Kansas, Oklahoma, Arkansas, Tennessee, Kentucky, Indiana, Illinois, Iowa, Georgia, Pennsylvania, New York, and North Carolina according to the Centers for Disease Control and Prevention (CDC; https://www.cdc.gov/heartland-virus/statistics/index.html). Studies have documented HRTV RNA in *A. americanum* ticks and HRTV-neutralizing antibodies in vertebrate animals in these states (*8*–*13*). However, the distribution of *A. americanum* ticks is wider and growing, possibly because of climate change, which could lead to HRTV range expansion (*3*,*11*). Of note, vertebrate animals with neutralizing antibodies to HRTV have been documented in states without confirmed human cases, including Texas, Florida, South Carolina, and Louisiana in the south and Vermont, New Hampshire, and Maine in the northeast (*12*,*13*). To date, no seropositive animals have been reported from Maryland or Virginia in the mid-Atlantic region. We describe a fatal human case of HRTV infection with secondary HLH in which initial infection likely occurred in either Maryland or Virginia.

## The Study

The patient was a man in his late 60s who had a medical history of splenectomy from remote trauma, coronary artery disease, and hypertension. He was seen at an emergency department in November 2021 for 5 days of fever, nonbloody diarrhea, dyspnea, myalgias, and malaise. At initial examination, he appeared fatigued but was alert and oriented. Laboratory results were notable for hyponatremia, mildly elevated liver enzymes, leukopenia, and thrombocytopenia (Table). The patient had homes in rural areas of Maryland and Virginia and had not traveled outside of this area in the previous 3 months. He spent time outdoors on his properties but did not recall attached ticks or tick bites. Despite the lack of known tick bites, the symptom constellation and potential exposure led clinicians to highly suspect tickborne illness; they prescribed doxycycline and discharged the patient home.

**Table:** Laboratory findings in a fatal case of heartland virus disease acquired in the mid-Atlantic region, United States*

Test	Reference range	Days after symptom onset				
		5	7	9	11	13
Temperature, °C	35.5–38.3	36.8	38.5	38.5	39.1	36.9
Blood cell counts						
Leukocyte count, × 10^3^ cells/μL	4.50–11.00	2.4	3.7	3.5	2.48	3.25
Absolute neutrophil count, cells/μL	1.50–7.80	ND	ND	ND	0.99	0.88
Absolute lymphocyte count, cells/μL	1.10–4.80	ND	ND	ND	1.22	1.76
Hemoglobin, g/dL	13.9–16.3	14.5	14.9	14.6	14.4	11.7
Platelets, × 10^3^/μL	150–350	178	106	82	59	61
Blood chemistry test results						
Sodium, mmol/L	135–148	126	115	120	129	136
Potassium, mmol/L	3.5–5.1	3.7	3.3	4.2	4.4	4.4
Carbon dioxide, mmol/L	21–31	22	22	20	17	18
Anion gap, mmol/L	7–16	13	15	12	12	11
Blood urea nitrogen, mg/dL	7–22	12	12	16	26	68
Creatinine, mg/dL	0.6–1.3	1.0	1.3	1.2	1.4	4.4
Aspartate aminotransferase, units/L	<37	45	359	434	590	617
Alanine aminotransferase, units/L	<40	46	238	262	209	156
Alkaline phosphatase, units/L	30–120	69	64	53	67	89
Cerebrospinal fluid test results						
Leukocyte count, cells/mm^3^	0–5	ND	2	ND	ND	ND
Glucose, mg/dL	40–70	ND	80	ND	ND	ND
Protein, mg/dL	12–60	ND	58	ND	ND	ND
Cardiac test results						
Troponin I, ng/mL	<0.04	ND	ND	ND	0.21	0.38
Troponin T, high sensitivity, ng/L	0–19	14	22	30	ND	ND
Pro-BNP, pg/mL	5–125	ND	ND	ND	4,258	ND
Lipid panel test results						
Cholesterol, total, mg/dL	<200	ND	ND	ND	69	ND
Triglycerides, mg/dL	<150	ND	ND	ND	147	ND
Other test results						
D-dimer, mg/L	0.00–0.49	ND	ND	2.72	3.71	ND
Creatine kinase, U/L	24–195	ND	ND	ND	8,727	11,083
Lactic acid, mmol/L	0.5–2.0	1.6	1.8	ND	2.5	2.4
Lactate dehydrogenase, U/L	118–273	ND	ND	1,412	1,709	ND
Ferritin, ng/mL	30–400	ND	ND	ND	47,445	174,957
Fibrinogen, mg/dL	170–422	ND	ND	199	224	170
C-reactive protein, mg/dL	<0.5	ND	1.2	0.6	0.5	ND
Interleukin 2 receptor, pg/mL	532–1,891	ND	ND	ND	9,390	ND

*BNP, B-type natriuretic peptide; ND, not done.

Two days later, on day 7 after symptom onset, the patient returned to the emergency department with confusion, an unsteady gait, and new fecal and urinary incontinence; he was admitted for inpatient management. He had progressive encephalopathy with hyponatremia and rising transaminases (Table). Results of neurologic workup and imaging were unremarkable (Table). Computed tomography imaging of the abdomen and pelvis showed new pelvic and inguinal lymphadenopathy. The patient was treated with hypertonic saline, intravenous doxycycline, and piperacillin/tazobactam.

Because of clinical deterioration, he was transferred to a tertiary care center. At arrival at the tertiary center, he was fatigued and disoriented. Physical examination demonstrated new hepatomegaly and lower extremity livedo reticularis. Results of broad testing for infectious etiologies was negative (Appendix Table). Laboratory results demonstrated increased creatine kinase (9,567 U/L), lactate (2.5 mg/dL), lactate dehydrogenase (1,709 U/L), and ferritin (47,445 ng/mL). Interleukin 2 receptor, a marker for HLH, was also elevated (9,390 pg/mL) (Table). Immunosuppressive agents for management of likely secondary HLH were deferred while clinicians conducted a diagnostic work-up of the underlying disease process. An arboviral disease was the leading diagnostic consideration, but limited availability of commercial diagnostic testing for tickborne diseases delayed diagnosis.

The patient’s clinical course continued to deteriorate. He had acute respiratory failure, renal failure, and a cardiac arrest. He was transitioned to comfort care and died on day 13 after symptom onset.

**Figure 1:**
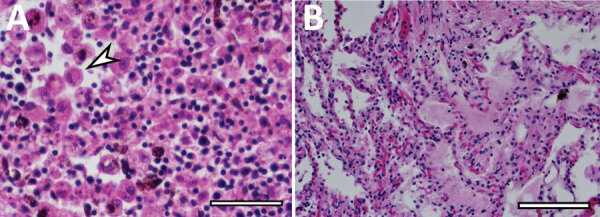
Postmortem autopsy findings in a fatal case of heartland virus disease acquired in the mid-Atlantic region, United States. A) Hematoxylin and eosin stain of patient accessory spleen; arrow indicates congestion with hemophagocytic histiocytes. Scale bar indicates 50 μm. B) Hematoxylin and eosin stain showing pulmonary hyperinflammation, including pleural thickening and adhesions, and pulmonary fibrosis, edema, and calcifications. Scale bar indicates 125 μm.

**Figure 2:**
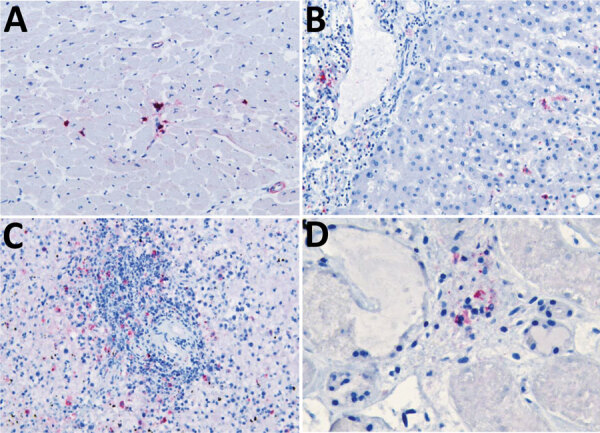
Viral immunostaining of samples from a fatal case of heartland virus disease acquired in the mid-Atlantic region, United States. Heartland virus antigen was detected in multiple organs. A) Mononuclear interstitial inflammatory cell of myocardium. Original magnification ×20. B) Periportal macrophages and Kupffer cells in liver. Original magnification ×20. C) Large hematopoietic cells of spleen. Original magnification ×20. D) Inflammatory interstitial cells of kidney. Original magnification ×40.

Because of concern for arboviral illness, the Virginia Department of Health (VDH) initiated an investigation and sent a serum specimen to CDC for testing (Appendix). Quantitative reverse transcription PCR was notably positive for HRTV RNA (Appendix Table). Autopsy findings identified markedly congested accessory spleens with abundant histiocytes, phagocytosing erythrocytes, and pulmonary hyperinflammation (Figure 1). Immunohistochemistry testing of heart, spleen, kidney, and liver samples were positive for HRTV at CDC (Figure 2). Immunohistochemistry of the spleen was negative for Epstein-Barr virus (EBV) at the clinical institution. The autopsy report concluded that the cause of death was respiratory failure secondary to hyperinflammation due to HLH, likely triggered by HRTV infection.

VDH performed tick drags at the patient’s 2 properties in eastern Maryland and central Virginia during early- to mid-June 2022. VDH collected a total of 193 ticks across the properties, which were sent to CDC for testing (Appendix). The tick pools collected from both properties tested negative for HRTV RNA.

## Conclusions

HRTV disease has been reported in >50 patients in states across the midwestern and southern United States (*1*–*7*). A bite from an *A. americanum* tick is the only known means of environmental HRTV transmission (*1*). Corresponding to *A. americanum* tick seasonal activity, all reported cases have occurred during April–September, and symptoms developed during June in most case-patients (*1*,*3*). Because the incubation period for HRTV is estimated to be 2 weeks, this patient was likely infected in late October. Adult ticks are minimally active at that time; however, larval ticks can become infected with HRTV and can still be observed during October (*1*,*14*). We suspect this patient was bitten by larval ticks unknowingly because of their small size, and that the bite marks healed before his clinical signs and symptoms appeared.

Maryland and Virginia fall within the *A. americanum* tick distribution area, but we found no previous reports of HRTV illness from those states during a literature search, and CDC had no reported cases from those states. Among 193 ticks collected during tick drags of both properties, no HRTV-infected vectors were found, but this result does not exclude HRTV in either state. Previous studies report low overall minimum infection rates among *A. americanum* ticks from other states, ranging from 0.4 to 11/1,000 ticks (1 infected tick/90–2,174 collected) (*1*,*8*,*10*,*11*). We suspect the Virginia property was the likely location of infection, based on the number of ticks VDH collected while sampling an area that the patient frequented 10–14 days before symptom onset and because fewer ticks were collected from the Maryland property (Appendix).

The patient’s clinical and laboratory findings were consistent with HLH secondary to HRTV infection. HLH has been documented in several cases of infection with the related *Bandavirus*, severe fever with thrombocytopenia syndrome virus, and in at least 1 case of HRTV infection (*1*,*4*). Reports showed corticosteroids and ribavirin did not effectively treat severe fever with thrombocytopenia syndrome–triggered HLH, but preliminary clinical data shows potential benefit from favipiravir (*1*,*15*). Currently, clinical management for HRTV infection is supportive care (*3*).

We hypothesize that HRTV infection is underrecognized and mainly diagnosed when severe disease leads to additional testing at referral centers. Although lack of responsiveness to appropriate antimicrobial agents for bacterial tickborne illness might suggest severe disease (*2*), self-limited disease likely is undiagnosed or diagnosed as another tickborne disease. Because tick ranges are increasing overall, incidence of previously regional tickborne infections, such as HRTV, likely will continue to increase. Expanding testing capabilities for arbovirus and tickborne infections, including multiplex testing, would enable real-time assessment and management of patients with potential arboviral and other tickborne infections.

Appendix Additional information on a fatal case of heartland virus disease acquired in the mid-Atlantic region, United States.
