# Supplementary material for: Fatal Case of Heartland Virus Disease Acquired in the Mid-Atlantic Region, United States
Source: Emerg Infect Dis. 2023 May;29(5):992–6. doi: 10.3201/eid2905.221488 (PMC10124632; doi:10.3201/eid2905.221488)
Supplement: Appendix — Additional information on a fatal case of heartland virus disease acquired in the mid-Atlantic region, United States. [file 22-1488-Techapp-s1.pdf]

Article DOI: https://doi.org/10.3201/eid2905.221488

Fatal Case of Heartland Virus Disease Acquired in the Mid-Atlantic Region, United States

Appendix

CDC Serology Testing

Given the severity of the illness, fatal outcome, and the fact that symptoms were consistent with tickborne arboviral illness, the Virginia Department of Health (VDH) initiated an investigation and sent a serum specimen obtained during laboratory testing before death to the Centers for Disease Control and Prevention (CDC) Arboviral Diseases Branch in Fort Collins, Colorado for testing. RT-qPCR was negative for Bourbon viral RNA but positive for heartland virus (HRTV) RNA and HRTV was isolated. IgM serology was negative for Powassan virus.

Tick HRTV Testing

To determine the likely location where the patient acquired HRTV and inform public health measures, the VDH performed tick drags using standard methods (*1*) at the patient's two properties in eastern Maryland and central Virginia in early- to mid-June 2022. At the Maryland property, the landscape was well maintained. Surveyors were not able to collect any ticks via their tick drags on the manicured, high human traffic portion of the property. Ticks that were collected were from a minimally accessible overgrown area of the property. In total, the survey covered 860 m^2 and yielded 31 nymph and 7 adult stage *A. americanum* ticks. The central Virginia farm was noted to be of forest and field environment and more readily yielded ticks. Tick drags were performed along the property, including a trail within the farm that the patient frequented 10–14 days before symptom onset. In total, the survey covered 1,620 m^2 and yielded 134 nymph and 15 adult *A. americanum* ticks, as well as 6 adult *Haemaphysalis longicornis* ticks.

Tick pool homogenization, RNA extraction, and viral screening were performed by RT-qPCR with previously described protocols (*2*). Tested adult tick pools ranged from 1–5 ticks in size, and nymph tick pools ranged from 6–25 ticks per pool. None of the tick pools collected from either property tested positive for HRTV RNA.

Immunohistochemistry

CDC Infectious Diseases Pathology Branch (IDPB) received formalin-fixed, paraffin-embedded samples from heart, spleen, kidney, and liver and conducted an immunohistochemical assay for HRTV using a rabbit polyclonal serum raised against HRTV nucleocapsid protein, as previously described (*3*), at 1:1,000 dilution and using a Mach 4 Universal AP Polymer Kit (Biocare Medical) with Permanent Red Chromogen (Cell Marque/Millipore Sigma).

References

1. Brinkerhoff RJ, Gilliam WF, Gaines D. Lyme disease, Virginia, USA, 2000–2011. Emerg Infect Dis. 2014;20:1661–8. PubMed https://doi.org/10.3201/eid2010.130782

2. Savage HM, Godsey MS, Lambert A, Panella NA, Burkhalter KL, Harmon JR, et al. First detection of heartland virus (Bunyaviridae: Phlebovirus) from field collected arthropods. Am J Trop Med Hyg. 2013;89:445–52. PubMed https://doi.org/10.4269/ajtmh.13-0209

3. McMullan LK, Folk SM, Kelly AJ, MacNeil A, Goldsmith CS, Metcalfe MG, et al. A new phlebovirus associated with severe febrile illness in Missouri. N Engl J Med. 2012;367:834–41. PubMed https://doi.org/10.1056/NEJMoa1203378

Appendix Table. Infectious disease testing for patient with Heartland virus, mid-Atlantic, USA*

Test	Result
Viral	
Influenza A/B PCR, NP swab	Negative
SARS-CoV-2 PCR, NP swab	Negative
Respiratory viral panel, NP swab	Negative
HAV IgM, serum	Negative
HBV Core IgM + Hbs Ag, serum	Negative
HCV antibody, serum	Negative
CMV PCR, CSF	Negative
Enterovirus PCR, CSF	Negative
HSV1/2 PCR, CSF	Negative
HHV6 PCR, CSF	Negative
Varicella-zoster virus PCR, CSF	Negative
Human parechovirus, PCR, CSF	Negative
HIV1/2 antigen + antibody, serum	Negative
CMV PCR, serum	Negative
**Epstein-Barr virus PCR, serum**	**Viral load 1,280**
Varicella-zoster virus PCR, serum	Negative
Fungal	
Cryptococcus neoformans PCR, CSF	Negative
Fungal smear, blood	No hyphae
Beta D-glucan, serum	<31 (negative)
Galactomannan, serum	0.13 (negative)
Histoplasma antigen, urine	Negative
Histoplasma antibodies, serum	Negative
Blastomyces antibodies, serum	Negative
Coccidioides total antibodies, serum	Negative
Vector-borne and zoonotic	
Lyme IgG + IgM, serum	Negative
Babesia microti antibodies, serum	Negative
Erlichia panel PCR, serum	Negative
Rickettsia SFG IgM, serum	Negative
Rickettsia SFG IgG, serum	Positive
West Nile Virus IgM, serum	Positive
Anaplasma phagocytophilum PCR, serum	Negative
Coxiella burnetii IgG + IgM, serum	Negative
Leptospira PCR, serum	Negative
Bourbon virus PCR, serum	Negative
Powassan virus IgM, serum	Negative
**Heartland virus PCR, serum**	**Positive**
Bacterial	
Escheria coli K1 PCR, CSF	Negative
Haemophilus influenzae PCR, CSF	Negative
Listeria monocytogenes PCR, CSF	Negative
Neisseria meningitidis PCR, CSF	Negative
Streptococcus agalactiae PCR, CSF	Negative
Streptococcus pneumoniae PCR, CSF	Negative
Legionella pneumophila antigen, urine	Negative
Aerobic and anaerobic culture, blood	No growth
Culture, urine	No growth

*Bold text indicates positive results. CMV, cytomegaly virus; CSF, cerebrospinal fluid; HAV, hepatitis A virus; HBV, hepatitis B virus; HCV, hepatitis C virus; HHV6; human herpes virus 6; HSV, herpes simplex virus; NP, nasopharyngeal.
